# Chemical Versus Germ Theories of Disease

**Published:** 1885-06

**Authors:** F. R. Campbell


### BUFFALO

### Medical and Surgical Journal.

Vol. XXIV. JUNE, 1885. No. 11.

### Original Communications.

### Chemical versus Germ Theories of Disease.

By F. R. Campbell, A. M., M. D.

In all fields of speculative thought, where demonstration has hitherto been impossible, we observe that hypotheses directly opposite in their tendencies have prevailed among scientists and philosophers. In mental science, idealism and materialism have alternately held sway. In medicine, the same phenomenon may be traced out. There was humoral pathology of the Ancients, essentially a chemical theory of disease, replaced by the vegetable parasitic hypothesis of Vallisneri, Hauptmann, and Linnæus. Then Stahl proposed a purely chemical theory of disease, but this has been followed by the germ theory, suggested by the revelations of the microscope, and brought to its present stage of development by such men as Virchow, Pasteur, Tyndall, and Koch. At present the germ theory has the strongest hold upon both the professional and popular mind. The discovery of each new so-called pathogenic micro-organism is hailed by the profession as a great addition to our knowledge of disease, while the people look upon a specimen of "comma bacillus" with as much interest as they would upon a *cobra de capello.* When Pasteur brought forth the results of his experiments on anthrax, pathologists were immediately converted into germ hunters, until now we have a microbe for nearly all febrile and contagious diseases; while specific germs of epidemic delusions and "bacillo-mania" will undoubtedly soon be imprisoned beneath the objectives of our microscopes. When the germ theory becomes an established fact, the true physician will become a mere manipulator of germicides, and the pathologist a student of microscopic botany.

But there are many honest scientific men who are unwilling to accept, in their present state, the views of the "bacteriologists," based almost entirely upon analogical and *post hoc ergo propter hoc* arguments. The powers of the microscope have almost reached their limit, but organic chemistry is as yet in its infancy, and it is the test tube and crucible, not the microscope, which will, in the future, make the greatest revelations in medical science. The recent investigations of chemical ferments and ptomaines have done much to turn again the tide of medical thought, and *contagia chemica* now rival the *contagia viva.*

It is the object of this paper to present, briefly, some of the objections to the germ theory of disease, and to bring forward a few arguments in favor of the chemical theory, neither approving the statement of the old physician who thought the world would be better off if Koch would turn his attention from the culture of germs to the cultivation of cabbages, nor agreeing with those wild theorists who would discover a germ as the source of every human ailment.

The germ theory, as adopted by most of its advocates, assumes that there is a specific micro-organism for each zymotic disease; that this micro-organism is capable of reproduction, and always produces similar chemical and anatomical changes in the body attacked. It is assumed that the organisms discovered are morphologically distinct, though it is, in many cases, impossible to differentiate them with our present means of investigation.

It is claimed that the existence of microbes as a cause of anthrax, tuberculosis, and, probably, cholera Asiatica, has been demonstrated, and by a perfectly logical deduction we may assume that germs are the cause of all infectious diseases. It is claimed that if a recognized variety of micro-organism is invariably found in the blood or tissues in connection with special disease; if these microbes are capable of cultivation outside of the body; if the products of these successive cultures, on inoculation, produce the original disease, and if, in the body thus inoculated, similar germs are found, it is demonstrated that these germs are the cause of the disease.

Many and able have been the arguments and experiments to prove this hypothesis, but it will not be necessary to present this side of the question, as all are familiar with the arguments in its favor. In spite of the attractiveness and plausibility of this theory, there are many objections in the way of its acceptance, which, to me, at least, are insuperable. Indeed, it is feared that the most earnest advocates of the germ theory cannot overcome these objections, unless, like the old Scotch professor of theology, "They look the difficulties square in the face and pass on."

1. These organisms are so minute that in the majority of cases it is impossible to differentiate microbes, which, if we accept the germ theory, produce very different results. No one can distinguish the micrococcus diphtheriticus from the micrococcus vacciniæ, nor can the gonococcus be differentiated from the micrococcus ureæ, though the former is supposed to produce urethral inflammation, while the latter confines its energies to the manufacture of carbonate of ammonia from urea.

2. It is very questionable if the micrococci of vaccine virus have anything to do with its action. Even filtered vaccine lymph has been known to be efficacious, though this has been denied by some authorities. Dr. Hartshorne claims that filtration may produce a chemical change in unstable compounds, so that it is possible that, even when we have well-marked microorganisms in a fluid which loses its specific character after their removal, the neutralization may be due to a chemical change. On the other hand, Hallier has shown that even pathogenic micro-organisms, when thoroughly washed, may be introduced into the system without producing disease, showing that there must be a chemical substance in connection with the bacteria which produces the pathological changes.

3. Bacteria and micrococci are always found on the surface of mucous membranes, and these organisms differ in no respect from those found in disease. According to some authorities, micrococci are always present in the blood of healthy persons. Koloczek, Richardson, Emmerich, Sternberg, and Lauder Brunton claim that even the dreaded "comma bacillus" is normally present in the intestines, Brunton proving that they greatly increase in numbers when the nerve supply of the intestine is cut off. Lewis, of this country, claims that it is normally present in the mouth.

4. It has been proved by Burdon-Sanderson and Cohen that bacteria derive their nitrogen, not from the living tissues of the body, but from ammonia, a product of decomposition, hence we must admit that the decomposition precedes the development of the bacteria. Unless we accept the views of the chemist of Laputa, who believed that excrement could be converted into food, we cannot understand how bacteria can live upon their own excretions. Moreover, bacteria are vegetables, and there are many reasons for believing that, like other vegetables, they destroy substances injurious to animal life. The river Seine, contaminated by the sewage of Paris, is purified by the numerous plants which flourish in the stream. Dr. Waring gives the results of experiments made by the French Horticultural Society, showing how plants will purify contaminated water. Now, since bac teria are vegetable organisms, is it not reasonable to suppose that they are curative and not causative elements of disease? "Where the carrion is, there do the young eagles gather," said the prophet. Where there has been decomposition, there will you find bacteria. As an argument in favor of this view of the function of micro-organisms, we may mention the fact that in cases of septicæmia and in dead bodies the poison is most active and virulent in the earlier stages of disease or decomposition, before the bacteria are most numerous. It is upon this fact that Prof. Owen bases his theory that micro-organisms are the scavengers of the human body.

5. Suppuration will occur when there are no micro-organisms present (Orthmann). On the other hand, bacteria have been found under Lister's dressings when there was no suppuration. Paul Bert and Rosenberger have destroyed all the bacteria in a septic fluid without diminishing its septic character. Griffini has found that saliva and urine, when deprived of their micro-organisms, will produce septicæmia in rabbits, all of which facts go to prove that bacteria are not the cause of septicæmia.

6. It is claimed by some of the most eminent observers, Buchner, Nägeli, Bastian, Billroth, and Sanderson, that the species of bacteria are convertible, one into another having entirely different properties. Sanderson says that "the influence of environment upon organisms such as bacteria is so great that it appears as if it were almost paramount." May it not be true, then, that the form of many of these organisms depends largely upon the surrounding medium? To quote from Ziegler: "The researches of Koch and his pupils do not prove that the qualities of the bacteria examined by them are perfectly constant. They only show that the morphological and physiological qualities possessed by a bacterium are retained with some tenacity. On the other hand, the researches of Nägeli, Buchner and Wernich seem to afford evidence that this constancy is not shown under all conditions. Changes of nutrient medium may have some effect on the form and size of the cells, on their mode of multiplication, and on their fermentative and physiological properties." If we admit that the morphology of bacteria depends partly upon the surrounding medium, we cannot possibly deny the validity of Buchner's experiments showing how the hay bacillus could be converted into the bacillus anthracis, and conversely.

7. If we cannot accept the theory of the mutability of bacteria, it is quite reasonable to suppose that special micro-organisms select special pathological lesions as a favorable culture ground. Just as particular trees select a soil adapted to their wants, so may the specific organisms locate in special diseased tissues, the bacillus tuberculosis flourishes in tubercle, the comma bacillus in the intestine, and micrococci in a diseased throat. If this is true, specific micro-organisms will have only a diagnostic significance.

8. These micro-organisms are not uniformly present. Klein states that bacteria are sometimes absent in typhoid fever. The bacillus tuberculosis may be accidental only, for—

(*a*) The contagiousness of tuberculosis is extremely doubtful.

(*b*) Tuberculosis has been induced by the inoculation of irritating substances not containing bacilli (Schotellius, Wood, Sanderson, Formad and Lebert).

(*c*) The bacilli of other affections, *e. g.*, syphilis, when inoculated in animals, will produce a disease resembling tubercle.

(*d*) Tuberculous deposits do not always contain bacilli, as has been shown by Sternberg, Spina, and Prudden, the last mentioned making six hundred and ninety-five sections from ninety-five tubercles of a tuberculous pleura, using all of Koch's precautions, and failing to discover the bacillus, though he has seen it in other cases. Koch gets over this difficulty by claiming that his bacillus is alone pathognomonic of tubercle. But this is begging the whole question. When the bacillus tuberculosis was found in various tissues, it was at once assumed that these tissues were all tuberculous, until now we have tuberculous abscesses, bones, muscles, testes, and tuberculous brains. With this sweeping assertion, a host of surgical diseases, formerly supposed to be distinct, were brushed into the tuberculous class.

9. If we assume that micro-organisms are the cause of all zymotic diseases because they accompany fermentation, we can, with equal propriety, assume that microbes are the source of all obscure reactions in organic chemistry. There should be a microbe in the salivary glands and pancreas to convert starch into sugar; a microbe in the stomach to change albuminoids into peptones; in fact, a micro-organism in each gland to bring about the chemical changes produced therein. Furthermore, even if these minute organisms do produce some chemical changes, it is not necessary to assume that they are specific. Simple addition or abstraction of oxygen is all that is required in most of them, and whether in a preparation of starch, for example, we use saliva, vegetable diastase or sulphuric acid, the result is the same. According to Ziegler, the same bacteria will produce lactic acid in milk and ammoniacal decomposition in meat. Brieger claims that the various aromatic products of the putrefaction of albumen are equally well obtained, whether it is set up by the addition of sewer mud or pancreas.

10. It is impossible to separate a micro-organism entirely from the medium in which it exists. In repeated cultures of bacteria this medium is only diluted, and it may be that the diminished toxic effect is due to the dilution of the poison, and not to the benign modification of the organism. Moreover, there is some evidence to prove that a chemical ferment can increase in volume as well as a bacterial ferment.

11. The specific character of no micro-organism, except, possibly, the bacillus anthracis, has been demonstrated. It seems very improbable that the comma bacillus, which now claims so much attention, is not the cause of cholera, for—

(*a*) Dr. Strauss, of the French Cholera Commission, reports that "the shorter and more violent were the fatal attacks of cholera, the fewer were the bacteria found in the intestine." This is the very opposite of what we should find were cholera due to bacteria.

(*b*) Koch, after showing how rapidly "comma bacilli" multiply when moist, states that they die after drying more quickly than almost any other form of bacteria. "As a rule, even after three hours' drying, every vestige of life disappears." But there are facts to prove that the fomites of cholera have preserved their infectious properties in dry places.

(*c*) No one has been able to produce cholera in any animal by inoculating "comma bacilli." Prof. Klein has even swallowed them without any injurious result. Pigs inoculated with cholera dejecta die, according to Emmerich, with septicæmia, with no choleraic symptoms whatever.

(*d*) Lastly, the "comma bacilli" have been discovered only in the intestine. Koch assumes that they secrete a poison which produces the symptoms, but Emmerich and Brunton have shown that the choleraic intestine is incapable of absorbing, and that reaction in the disease is too rapid to admit of a bacterial origin.

Having thus pointed out some of the difficulties to be overcome before accepting the germ theory of disease, we will now attempt to show how all the phenomena of zymotic affections may be at least equally well explained from a chemical point of view.

Chemical theories of disease assume that all diseases attended with variations of temperature are due to some abnormal chemical action in the system. In non-infectious febrile diseases the abnormal chemical action may be instituted by some unusual impression upon the nervous system, such as mental shock, surgical injury, or sudden change of temperature. Infectious and contagious diseases are due to chemical changes in the body, produced by the introduction of some chemical substance from without, presumably derived from another person having the same or a similar disease, and resembling the ferments produced by glandular action. As examples of the former class, may be mentioned pneumonia, meningitis simplex, and the variations in temperature attending concussion of the brain. We know that many chemical changes in the human body are regulated by the nervous system. Fear and anxiety will suppress the secretions of the gastric glands; the poisonous compounds of the saliva of men and animals are increased by anger; while diabetes mellitus may be induced by irritation of the floor of the fourth vertricle. The recent experiments of Italian chemists have demonstrated that poisonous alkaloids, or ptomaines, are constantly found in the body, being eliminated with the urine, sweat and fœces. When these compounds are formed more rapidly than they can be eliminated, disease is produced. Schelmi, Pesci and Vella have proved that alkaloids and peptones are produced during the putrefaction of albuminoids; Spica has shown that a poisonous alkaloid can be extracted from normal blood; while Brieger has discovered a poisonous substance found in connection with peptones in ordinary stomach digestion. It is quite probable that the retention or excessive formation of these poisons will produce various diseases, just as the retention of uric acid or oxalic acid in the system produce their respective diatheses.

But in zymotic diseases it is assumed that the chemical substances which sets up the changes in the system are introduced from without; that they have special affinities for particular tissues, and that they themselves can increase in volume and still remain unaltered in composition. At the first glance it seems doubtful if these phenomena can belong to any except a vitalized substance. Yet we will find, on reflection, that all these requirements can be answered without calling any germs to our assistance.

It is no more remarkable that the chemical contagium of mumps should select the parotid gland, or the contagium of measles, the mucous lining of the air passages for their seat of action, than that woorara should confine its activity to paralyzing the motor nerves, and hydrocyanic acid limit its action to the respiratory centre. Zymotic chemical poisons may have selective powers just as well as drugs or bacteria. It is not necessary to assume that the quantity of the contagious poison is large for its action in catalytic, or, preferably, eryptolitic, to use the word coined by Prof. R. A. Witthaus. It may induce chemical changes in other substances without itself undergoing change. Take, for example, the conversion of alcohol into ether by means of sulphuric acid, or the conversion of starch into sugar by the mineral acids, vegetable diastase or ptyalin. In all these cases the substance instituting the chemical changes is simply diluted and not otherwise altered. Moreover, some organic chemical compounds are capable of assimilating other substances. It is a well-known fact that a bad egg in a box will contaminate its neighbors. It is scarcely possible, however, that bacteria could penetrate the shell and vitelline membrane of the egg. Only chemical substances are capable of osmosis. Take, as another example, the poison of the *cobra* inoculated into an animal. The animal thus poisoned is destroyed and its secretions, when introduced into other animals, produce the same symptoms as the original poison, yet no one has attempted to show that the poison glands of the *cobra* secrete bacteria. So it is with the zymotic poisons; they produce pathological chemical changes and at the same time themselves increase in volume. In the case of septicæmia this has apparently been proved. Richardson and others have succeeded in separating a poisonous alkaloid in septic matter. This alkaloid produces, on inoculation, all the symptoms of septicæmia. It has been claimed by some observers that the inoculation of septin is followed by very different symptoms from the inoculation of septic pus. The former acts like a dose of an alkaloid, while the latter produces genuine septicæmia, due to the presence of the bacteria. But there are authorities, Paul Bert, Panum, Coze, Bergman, Schmiedeberg, Vulpian, Clementi, Zweifel, and many others, who have made experiments to prove that septicæmia may be produced without the presence of any organisms whatever. The alkaloid of septic matter is not the ferment, but the ferment is certainly not bacterial.

Zymotic poisons may be solid, liquid, or gaseous, thus accounting for the difference between contagious and infectious diseases, which can hardly be explained by the germ theory.

The poisons of purely infectious diseases are gaseous; the poisons of purely contagious diseases are solid or non-volatile liquids; the poisons of diseases both contagious and infectious are solids or liquids capable of volatilization. If these diseases are all alike due to micro-organisms, why are they not all alike contagious and infectious? And why should we not believe our patients when they tell us they have contracted gonorrhœa in water-closets? The attempts to explain these differences by the motility of bacteria have been failures.

The influence of season on disease can be more easily accounted for by assuming a chemical than a bacterial origin of disease. If germs were the cause of zymotic diseases we would expect to find them all most active and fatal in summer when micro-organisms are most readily developed. This, however, is not the actual case, for many zymotic diseases are most prevalent in cold weather.

With chemical substances, on the other hand, some require cold and others heat for solution. The phosphates found in urine, for instance, are precipitated by heat; the urates, by cold. So the chemical contagion may be affected by temperature; heat reducing some, like that of diphtheria, to a more solid state, and rendering others, like the poison of cholera, more volatile. The chemical constitution of the atmosphere varies in the different seasons. In summer there is more ammonia in the air than there is in winter. Many of the zymotic poisons seem to be carried, probably by solution, in the products of decomposition, such as ammonia and sulphuretted hydrogen, and this will account for the increased virulence of some zymotic diseases in badly drained and filthy districts.

In a similar manner we can explain nearly all the phenomena of zymotic diseases, by showing the analogy between the behavior of zymotic poisons and chemical compounds. We will discuss some of these phenomena in detail:

1. *Period of incubation.* In connection with the chemical processes associated with a zymotic disease, poisonous products are formed which are in part eliminated by the excretory organs. When these poisonous substances have accumulated in the system in sufficient quantities, the characteristic symptoms of the disease appear. The poisonous substance is produced more rapidly in some diseases than in others, thus explaining variations in the length of periods of incubation. In cholera Asiatica, for example, the poison is rapidly formed, and we have a very short period of incubation. In syphilis and hydrophobia, on the other hand, the chemical changes are slow and a long period of incubation is the consequence. The variation of the period of incubation of the same disease in different persons may be due to the chemical constitution of the body affected or to the activity of the excretory organs. If the excretory functions are active the poison accumulates slowly and the period of incubation is correspondingly lengthened. Variations in intensity of a disease may be accounted for in the same way. The immunity of some persons to zymotic diseases may be explained by the poison being formed in small quantities, or not at all, and being eliminated from the system as fast as produced. What physician has not observed the remarkably varied sequelæ of chancres? Some patients are rapidly overcome by the disease; others have a few papules and pustules, and that is all. There are many arguments to support the views of those who refuse to believe in the duality of syphilis. When the initial lesion is not followed by constitutional symptoms it may be due to the elimination of the poison from the system as fast as it is formed. Here the same poison is introduced and the various symptoms depend entirely upon the constitution of the patient inoculated.

The influence of hygienic surroundings is also explained on this theory. Impure air, dampness and filth affect the excretory functions of the lungs, skin and kidneys injuriously. As a consequence, typhus, scarlatina, measles and all infectious diseases are aggravated by such conditions.

2. *Sudden appearance of symptoms.* This is probably due to a cumulative effect of the poison formed. We know that many drugs, *e. g.*, digitalis and arsenic, may be administered for some time without producing any appreciable effect, when suddenly all the physiological symptoms of the medicine appear; and there is no good reason why the poison formed in disease may not act in a similar manner.

3. *Variations of temperature.* In all chemical reactions heat is either absorbed or liberated. As a rule, when substances pass from a solid to a liquid state heat is taken up; on passing from a liquid into a solid state heat is given off. When oxygen is given off from a compound, heat is absorbed. Example, the production of oxygen by heating chlorate of potash. When substances are oxidized heat is evolved. When fluids are formed and eliminated from the body in excess, as in Asiatic cholera, and in diseases attended with profuse perspiration, the temperature of the body is reduced. In Asiatic cholera there seems also to be deficient oxidation, for the blood rapidly becomes acid, excess of uric acid in the blood being supposed to indicate deficient oxidation, the fibrin diminishes, and urea is decreased. In rheumatism, on the other hand, the amount of fibrin in the blood is increased, and there is rise of temperature.

The albuminoids in the body pass through a long series of changes before being eliminated, and it is probable that some of these changes are attended with the absorption and others with the evolution of heat. So with the chemical changes in disease; the temperature of the body will rise or fall according as the prevailing chemical change is attended with the evolution or absorption of heat. Chemical reaction, attended with absorption of heat, seems to take place first in zymotic diseases, as in others, for we frequently have a chill and a reduction of temperature as the initial symptoms. Sometimes the secondary rise of temperature never takes place. There are cases of puerperal peritonitis, for example, in which the temperature remains below normal from the beginning.

4. *The eruption* on the skin, attending many zymotic diseases, is entirely analogous to that following the ingestion of many drugs. Belladonna causes a rash, which, according to the homœopaths, resembles that ot scarlet fever; the bromides and iodides produce pustular eruptions; shell fish and game, especially when "high," that is, when they contain post mortem alkaloids, will produce urticaria in some persons. So the poisons of contagious and infectious diseases bring about cutaneous changes; that of scarlet fever, like belladonna, manifesting the symptoms early; that of typhoid and syphilis, like the bromides, only after a considerable quantity exists in the system.

5. *The non-recurrence* of most zymotic affections may be explained, (*a*) by assuming that the chemical constituents of the tissues, which can be attacked by a given zymotic poison, have all undergone a change, so that there is nothing to be acted upon at a second exposure; (*b*) by the establishment of tolerance for a vigorous zymotic poison. It is a well-known fact that the doses of many medicines must be increased to produce constitutional effects. Tobacco, when first taken by a person, usually produces marked physiological symptoms, yet the system soon becomes accustomed to its presence, and then even large doses have but little effect. So with the zymotic poisons; even if the chemical constitution of the body were not changed by its first action, a tolerance might be established, which would prevent the recurrence of morbid symptoms. The human system has the power of adapting itself to the presence of poisons. The Styrians can take large quantities of arsenic without injurious results. People may become accustomed to the presence ot marsh miasms, without contracting malarial disease. This power, when once established, can be transmitted to posterity, for the natives of marshy districts seldom have ague, and the children of men who have had syphilis, even when they show no evidences of the inherited disease, are said to be exempt from the disease, or else will have it in a very mild form, a fact that has been observed in several parts of Portugal.

6. *The origin of zymotic poisons* may be in part accounted for by a chemical theory of disease. When organic matter undergoes decomposition, many compounds, probably alkaloidal in their nature, are produced. Water contaminated with decomposing vegetable substances will generally cause diarrhœa, especially in those persons not accustomed to its use. Some physicians think that typhoid fever may be brought about in the same manner. The effluvia from sewers will produce sore throat, and probably diphtheria. In three cases, during the past winter, the writer has seen malignant diphtheria developed in families who have removed from healthy localities into houses containing untrapped water-closets, though the other inhabitants of the block were unaffected. The "filth diseases," so called, all seem to be caused by the decomposition of organic matter. According to the germ theory, filth is only a favorable culture ground for the bacteria of these diseases, yet we frequently meet with zymotic affections where it seems impossible that the poison was derived from pre-existing cases of the disease.

Prof. Richardson goes so far as to believe that the zymotic poison may even be produced *de novo* in some individuals by powerful mental impression. In this way he accounts for cases of cholera, apparently due to fright. Although such an ideacannot be supported by scientific observation, it seems probable that cases of disease, apparently zymotic, may be developed under certain conditions, without the action of a specific poison.

7. *Mutability of zymotic disease.* We have seen that the same chemical substance, when introduced into different compounds, produces different results. Sulphuric acid in starch forms sugar; in alcohol, ether. Now, the chemical composition of the human body varies with conditions of age, disease, sex, and physiological state. Consequently the same zymotic poison may produce different diseases. The poison that will cause scarlet fever in a child may produce erysipelas in a wounded man, or puerperal fever in a lying-in woman. Measles and mumps usually prevail at the same time, the latter being most common in boys, while scarlatina anginosa and diphtheria can, in some cases, hardly be distinguished.

8. *Sepsis and antisepsis.* It has been shown that in the decomposition of the human body poisonous alkaloids are produced, those first formed being most virulent in their action. That is, the lower the state of oxidation, the more poisonous are the alkaloids formed. When pus has become septic, it contains an alkaloid, which, on injection into the system, will set up all the symptoms of septicæmia. This alkaloid has been called septin, and, although its exact composition is unknown, it is probably capable of further oxidation, thus being made innocuous. If a wound is cleaned perfectly and so dressed that but little air can come in contact with it, and so that the discharges can be rapidly removed, septin is formed in small quantities only, and is removed before much has been absorbed. If the so-called air dressing of the French surgeons is employed, the alkaloids are more highly oxidized, and are thus rendered harmless. Septicæmia seldom occurs in wounded animals, yet the wounds are freely exposed to the air. The only dressing is the scab which nature provides, and the only antiseptic employed is the tongue of the animal, which removes the putrefying substance. Here free exposure to the air seems to be nature's method of cure, that is, rapid oxidation of the products of putrefaction. Examine our best antiseptics, and what do you find? That they are either powerful oxidizing agents, like bichloride of mercury or permanganate of potash, or else they are elements having a strong affinity for organic matter, like chlorine and iodine. The former oxidize the septic poisons, converting them into other harmless alkaloids, the same process that is carried on more slowly by nature; the latter convert the alkaloids into inert compounds, probably muriates and iodides.

9. Lastly, chemical theories of disease afford a more rational basis of treatment than germ theories. Instead of seeking a suitable germ, which is powerless unless given in sufficient quantities to kill the patient, we will endeavor—

(*a*) To prevent the formation of the poison. Here prophylactic remedies prove serviceable, and for this reason we may give quinine and coffee to prevent malarial diseases; we use caustics to destroy the virus of the initial lesion of syphilis, and the homœopaths give belladonna to prevent scarlatina.

(*b*) We attempt to destroy or counteract the effects of the poison by giving chemical or physiological antidotes. Mercury apparently antidotes the effects of the syphilitic virus. Cinchona is our best remedy for malarial poisoning. We use oxidizing agents to destroy septic poison in a wound, and these same agents seem to alter the character of some inflammations, especially those of mucous membranes.

(*c*) We endeavor to eliminate the poison by promoting the activity of the excretory glands. The beneficial effects of a "sweat" in fever have long been recognized; the Hot Springs treatment is beneficial chiefly by its sudorific action. Diuretics will remove other poisons, while pure air promotes excretion by the lungs.

(*d*) Lastly, we endeavor to support our patient until the abnormal chemical processes cease, and the poison is eliminated from the system.

We have now given some of the analogies between zymotic poisons and the action of chemical substances. When organic chemistry is further developed, and scientists study pathological chemistry as well as pathological anatomy, we will expect our chemical hypothesis to be established as a fact. The labors of the bacteriologists are not, however, vain, for they will point out the relations between micro-organisms and abnormal chemical processes in the body, and perhaps by a process of *reductio ad absurdem* support the chemical theory of disease. They have already given the world many valuable discoveries. Just as the alchemists of old, while seeking the philosopher's stone and the elixir vitæ, brought forth what was even more valuable to mankind, modern chemistry, so the microscopists have paved the way for the greatest discovery of modern medicine, surgical antisepsis.

